# Severe Central Nervous System Co-infection With Streptococcus pneumoniae and Varicella-Zoster Virus Presenting as Meningoencephalitis

**DOI:** 10.7759/cureus.112261

**Published:** 2026-07-08

**Authors:** Tutul Chowdhury, Minhaz Murshad, Manisha Deb, Temilola O Akande, Aneek Ghosh, Nisha K Sapkota, Nafisa Hasna, Mohammad Zaman

**Affiliations:** 1 Pulmonary and Critical Care Medicine, Interfaith Medical Center, Brooklyn, USA; 2 Pulmonary and Critical Care Medicine, Brookdale University Hospital Medical Center, Brooklyn, USA; 3 Internal Medicine, Jersey Shore University Medical Center, Neptune, USA; 4 Pediatrics and Child Health, Sylhet MAG Osmani Medical College, Sylhet, BGD; 5 Internal Medicine, Brookdale University Hospital Medical Center, Brooklyn, USA; 6 Medicine, Brookdale University Hospital Medical Center, Brooklyn, USA; 7 Medicine, Interfaith Medical Center, Brooklyn, USA; 8 Medicine, Lake Erie College of Osteopathic Medicine, Erie, USA; 9 Internal Medicine, Interfaith Medical Center, Brooklyn, USA; 10 Critical Care Medicine, Brookdale University Hospital Medical Center, Brooklyn, USA

**Keywords:** central nervous system, co-infection, meningitis, streptococcus pneumoniae, stroke mimic, varicella zoster virus

## Abstract

Prompt identification of bacterial and viral central nervous system (CNS) infections is crucial; however, co-infection remains rare and diagnostically challenging. We report the case of a 61-year-old woman who presented with altered mental status and was initially suspected of having a stroke but was later diagnosed with severe sepsis and meningitis. Cerebrospinal fluid (CSF) analysis revealed elevated protein levels, and nucleic acid amplification testing (NAAT) identified concurrent *Streptococcus pneumoniae* and varicella-zoster virus (VZV) infection. Blood cultures subsequently confirmed *S. pneumoniae* bacteremia. Furthermore, the hospital course was complicated by metabolic encephalopathy, non-ST-segment elevation myocardial infarction (NSTEMI), and acute kidney injury (AKI). This case highlights the importance of maintaining a broad differential diagnosis and considering mixed CNS infections in atypical presentations while emphasizing the value of molecular testing. Early recognition and targeted therapy are essential for improving neurological outcomes in critically ill patients.

## Introduction

Bacterial meningitis remains a critical global health concern, with mortality rates reported as high as 54% [1-3]. In the management of central nervous system (CNS) infections, the primary objective is the rapid differentiation between bacterial and viral etiologies. *Streptococcus pneumoniae* remains the predominant causative pathogen, accounting for approximately 60% of cases [3,4], with antimicrobial non-susceptibility identified in 42.2% of isolates [2]. Although the incidence fluctuated during the COVID-19 pandemic, an 87% resurgence in pneumococcal meningitis was observed during 2022-2023, emphasizing the ongoing need for rapid identification and treatment of this pathogen [2]. While clinical diagnostic frameworks are typically designed to identify a single primary etiology, the simultaneous occurrence of bacterial and viral pathogens presents a significant diagnostic challenge. Such co-infections account for approximately 2.8%-4.8% of culture-positive meningitis cases in the pediatric population [3], although comparable data in adults remain limited. This phenomenon represents a complex clinical scenario in which multiple diagnostic categories must be considered simultaneously. Among viral etiologies, reactivation of varicella-zoster virus (VZV) predominantly affects older adults and immunocompromised individuals [4]. We present the case of a 61-year-old woman who developed acute metabolic encephalopathy precipitated by systemic sepsis secondary to meningitis caused by concurrent infection with *S. pneumoniae* and VZV.

## Case presentation

A 61-year-old woman with a history of hypertension presented with an acute stroke activation after being found down at her residence. According to the patient’s daughter, the patient was at her baseline state of health the evening prior to admission, at which time she developed acute encephalopathy characterized by severe agitation and aphasia. Upon arrival at the emergency department, the patient was in severe respiratory distress, with tachypnea (40 breaths/minute) and hypoxia, with an oxygen saturation of 87% on room air. She required immediate stabilization with non-invasive positive pressure ventilation (BiPAP) and intravenous sedation. On physical examination, the patient was obese, non-verbal, and responded only to noxious stimuli. Cutaneous examination was notable for facial erythema accompanied by retiform purpura, likely due to vasculitis. On detailed neurological examination, the patient was awake and alert and appeared to visually track and regard the examiner; however, she did not answer orientation questions, follow commands, or demonstrate any usable speech. She was unable to name, repeat, pantomime, or otherwise participate meaningfully in language testing. Cranial nerve examination revealed pupils measuring 4 mm that were round and equal, with minimal reactivity to light. Extraocular movements were intact without nystagmus, and the patient appeared to track the examiner appropriately. Blink-to-threat was present bilaterally. Jaw movements were intact. There was no facial asymmetry at rest or with spontaneous movement. Palatal elevation was symmetric. The tongue protruded in the midline with normal side-to-side movement, without evidence of atrophy or fasciculations. Head rotation and shoulder shrug were intact and symmetric (5/5 bilaterally). Motor examination demonstrated normal muscle bulk and tone throughout. The patient moved both upper extremities spontaneously against gravity. In the lower extremities, the legs fell to the bed when lifted; however, the patient demonstrated equal flexion bilaterally. There was no obvious asymmetry in spontaneous motor activity. Sensory examination demonstrated localization and withdrawal to painful stimuli in all four extremities. Coordination testing could not be performed because the patient was unable to understand or follow commands. The patient was noted to be using accessory muscles of respiration and appeared to be in respiratory distress.

Initial laboratory evaluation revealed profound leukocytosis, with a white blood cell count of 18,200/µL, and severe high-anion-gap metabolic acidosis secondary to hyperlactatemia. Laboratory values on admission, day 1, and day 14 are listed in Table 1.

**Table 1:** Lab results during admission and on day 1, day 7, and day 14

Labs	On admission	Day 7	Day 14	References range
Hemoglobin	13.4	10.5	9.8	11.0-15.0 g/dL
Hematocrit	43.6	33.6	29.3	35%-46%
White blood cell	18.2	7.0	10.8	4.5-10.2 x 10^6^/uL
Platelets	177	214	155	130-400 x 10^3^/uL
Troponin	2481	247.9	Not analyzed	5.0-19.7 ng/L
Glucose	123	132	64	80-115 mg/dL
Blood urea nitrogen	11	10	7	9.8-20.1 mg/dL
Creatinine	1.0	1.4	0.5	0.57-1.11 mg/dL
Sodium	140	142	138	136-145 mmol/L
Potassium	4.0	4.1	3.7	3.5-5.1 mmol/L
Chloride	105	105	104	98-107 mmol/L
Bicarbonate	14	24	24	23-31 mmol/L
Calcium	9.2	7.9	8.2	8.8-10.0 mg/dL
Albumin	3.3	2.8	2.8	3.2-4.6 g/dL
Magnesium	1.7	2.2	1.9	1.6-2.6 mg/dL
COVID-19 PCR	Negative	Not analyzed	Not analyzed	Negative
Prothrombin time	16.7	14.8	Not analyzed	9.8-13.4 sec
International normalized ratio (INR)	1.46	1.29	Not analyzed	0.85-1.15
Partial thromboplastin time (PTT)	27.9	24.9	Not Analyzed	24.9-35.9 sec
Thyroid-stimulating hormone	17.8	Not analyzed	Not analyzed	0.465-4.680 uIU/mL
Urine toxicology	Benzo positive	Not analyzed	Not Analyzed	Negative

**Figure 1:**
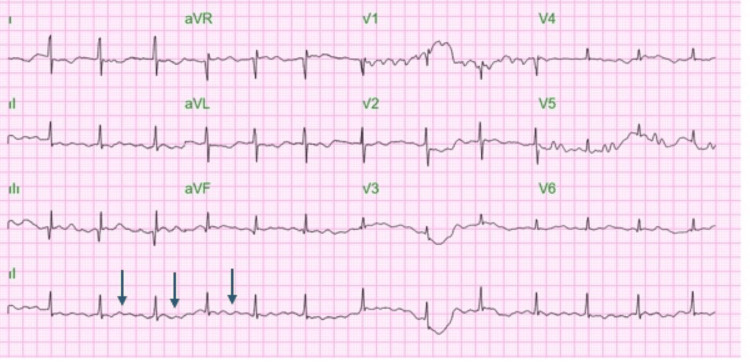
Electrocardiogram showing atrial flutter (saw tooth appearance; blue arrows)

An electrocardiogram (ECG) demonstrated atrial flutter (Figure 1), and high-sensitivity troponin was elevated at 2,481.1 ng/L, prompting a cardiology consultation for suspected type 2 non-ST-elevation myocardial infarction (NSTEMI). Urine toxicology was positive for benzodiazepines, which had been administered upon arrival in the emergency department for severe agitation. 

**Figure 2:**
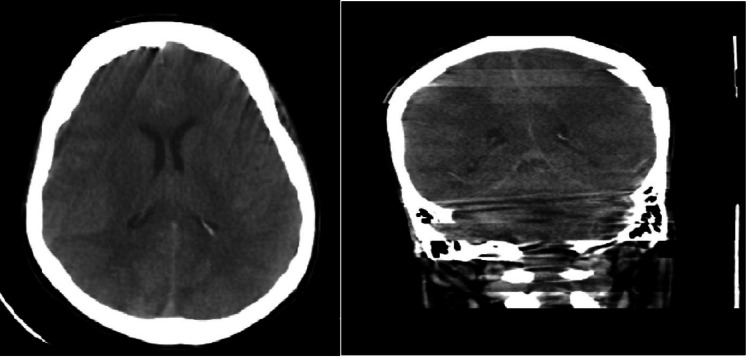
Computed tomography of the head showing no acute intracranial abnormalities

Given the clinical constellation of leukocytosis, severe lactic acidosis, and acute neurological deficits, the patient was initiated on empiric broad-spectrum antimicrobial therapy for suspected sepsis and admitted to the intensive care unit (ICU). At this juncture, although acute cortical cerebral infarction remained in the differential diagnosis, toxic-metabolic encephalopathy and meningoencephalitis were strongly considered the primary etiologies. Initial neuroimaging with non-contrast computed tomography (NCCT) of the head revealed no acute intracranial pathology (Figure 2). Subsequent computed tomography angiography (CTA) of the head and neck was non-diagnostic because of severe motion artifact.

**Figure 3:**
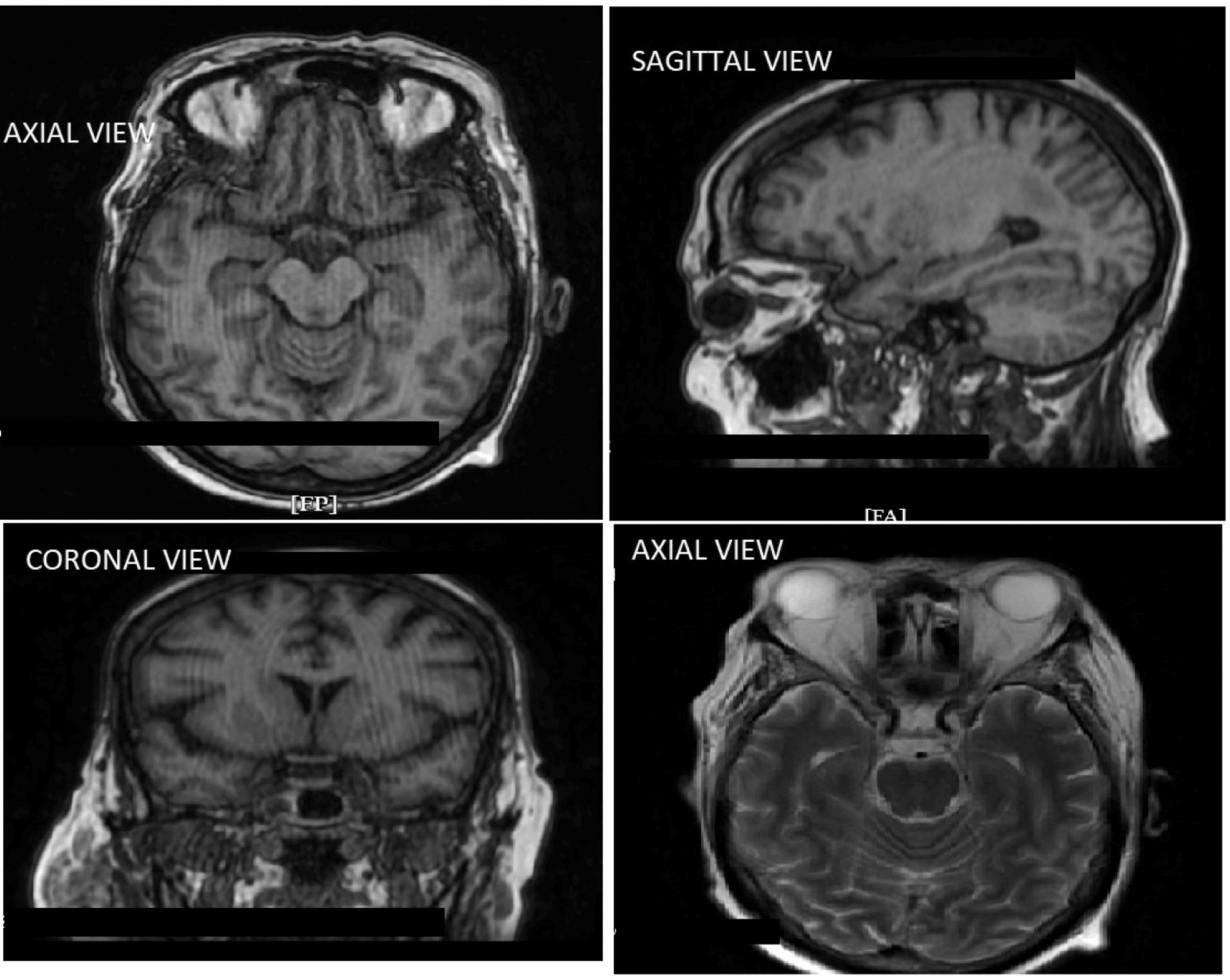
Non-contrast magnetic resonance imaging of the head showing no intracranial abnormalities

Non-contrast magnetic resonance imaging of the head was obtained, which was unremarkable (Figure 3).

Given the strong clinical suspicion for CNS infection, a lumbar puncture was performed, yielding cloudy, yellow CSF with an opening pressure of 30 cm H₂O (normal CSF opening pressure: 6-25 cm H₂O). Analysis revealed profound neutrophilic pleocytosis, with a white blood cell count of 12,870 cells/µL (97% neutrophils) and a red blood cell count of 1,650 cells/µL (Table 2).

**Table 2:** Cerebrospinal fluid analysis from lumbar puncture

Cerebrospinal fluid analysis labs	Value on initial lumbar puncture	Reference range
Appearance	Cloudy	Clear
Color	Yellow	Clear
Micro total protein	>600	12-45 mg/dL
WBC	12,870	<5 cells/uL
Neutrophil	97%	<6%
Monocyte	3%	15%-45%
RBC	1,650	0 cells/uL
Glucose	<12	50-80 mg/dL

While the CSF Gram stain demonstrated numerous white blood cells without visible organisms, a meningitis/encephalitis pathogen panel (Table 3) using nucleic acid amplification testing (NAAT) confirmed the simultaneous presence of *S. pneumoniae* and VZV. CSF culture showed no growth, with numerous white blood cells observed but no organisms seen on Gram stain. However, the patient had received antibiotics prior to the lumbar puncture, which may have reduced the yield of both the culture and Gram stain. Confirmatory VZV polymerase chain reaction (PCR) testing was negative.

**Table 3:** Meningitis/encephalitis pathogen panel performed on cerebrospinal fluid

Pathogen	Results	Reference range
*Escherichia coli*	Not detected	Not detected
Haemophilus influenzae	Not detected	Not detected
Listeria monocytogenes	Not detected	Not detected
Streptococcus agalactiae	Not detected	Not detected
Streptococcus pneumoniae	Detected	Not detected
Herpes simplex virus type 1	Not detected	Not detected
Herpes simplex virus type 2	Not detected	Not detected
Human herpesvirus 6	Not detected	Not detected
Human parechovirus	Not detected	Not detected
Varicella-zoster virus	Detected	Not detected
Cytomegalovirus	Not detected	Not detected
Enterovirus	Not detected	Not detected
*Neisseria meningitidis* (encapsulated)	Not detected	Not detected

These findings correlated with the blood cultures, which grew Gram-positive cocci in pairs and chains that were subsequently identified as *S. pneumoniae*.

On hospital day 2, the patient remained hemodynamically stable but required ongoing vasopressor support with norepinephrine and vasopressin. Her respiratory status showed interval improvement, allowing transition from BiPAP to a high-flow nasal cannula with an FiO₂ of 70%, while maintaining a low threshold for intubation. She demonstrated mild improvement in mental status, becoming awake and responsive, although she remained lethargic. Empiric antimicrobial therapy included ceftriaxone 2 g every 12 hours, vancomycin 1.5 g every 12 hours, and ampicillin 2 g every 4 hours, along with adjunctive dexamethasone 10 mg every 6 hours. Following pathogen identification, the antibiotic regimen was narrowed to intravenous ceftriaxone 2 g every 12 hours. Dexamethasone, which had been initiated before ceftriaxone, was continued at 10 mg every 6 hours for 4 days, while vancomycin and ampicillin were discontinued after blood culture confirmation. Bedside transthoracic echocardiography revealed reduced left ventricular systolic function (30%-50%) and a small pericardial effusion. She was managed with anticoagulation, initially with a heparin infusion and later transitioned to therapeutic enoxaparin, along with dual antiplatelet therapy and statin therapy. On hospital day 3, the patient was successfully weaned off all vasopressor support, allowing removal of the femoral central venous line. Her respiratory status continued to improve, with the FiO₂ requirement on high-flow nasal cannula reduced to 50% based on stable arterial blood gas results. Neurologically, she became more interactive and oriented and subsequently passed a bedside swallow evaluation, allowing initiation of a pureed diet. Levetiracetam (500 mg twice daily) was initiated for seizure prophylaxis based on neurocritical care recommendations. No seizure activity was detected on electroencephalography during the hospital stay, and close neurological monitoring was continued. However, her course was complicated by reduced urine output and a positive fluid balance, indicating the development of acute kidney injury, characterized by an increase in serum creatinine from 1.0 mg/dL to 1.4 mg/dL. A discrepancy in CSF protein values prompted repeat analysis, which confirmed markedly elevated CSF protein levels (>600 mg/dL), further supporting the diagnosis of *S. pneumoniae* meningitis. To confirm VZV infection, PCR testing was performed. Based on the recommendations of the infectious disease team, ceftriaxone was continued for 14 days, and acyclovir (10 mg/kg every 8 hours) was initiated for the treatment of VZV infection. Following completion of treatment, the patient was discharged home without any residual neurological deficits.

## Discussion

We present a rare case of community-acquired meningitis caused by concurrent *S. pneumoniae* and VZV infection, complicated by septic shock, acute metabolic encephalopathy, NSTEMI, and AKI. *S. pneumoniae* remains the leading cause of adult bacterial meningitis and is associated with substantial mortality despite advances in antimicrobial therapy and vaccination [2,3]. Although bacterial meningitis is a medical emergency requiring rapid diagnosis and treatment, the simultaneous detection of bacterial and viral pathogens within the CNS remains uncommon. The increasing use of NAATs and multiplex PCR panels has improved recognition of these mixed infections, particularly in critically ill patients with atypical presentations [4,5].

VZV is increasingly recognized as a cause of viral meningitis and meningoencephalitis in older adults. Importantly, CNS infection may occur in the absence of the characteristic dermatomal rash, potentially delaying diagnosis [6,7]. In our patient, severe pneumococcal sepsis may have contributed to reactivation of latent VZV through immune dysregulation associated with critical illness. Although bacterial-viral coinfection has been described in pediatric populations, adult cases remain rarely reported [6,7]. Our patient's presentation was atypical, with altered mental status and focal neurologic deficits in the absence of fever, prompting activation of a stroke code. The presence of blood in the cerebrospinal fluid further complicated the initial diagnostic evaluation. This case highlights the limitations of relying solely on the classic triad of fever, neck stiffness, and altered mental status, which is absent in many patients with bacterial meningitis. Clinicians should maintain a broad differential diagnosis in patients presenting with encephalopathy and focal neurologic deficits, particularly when accompanied by laboratory evidence of systemic inflammation.

The CSF profile was strongly suggestive of bacterial meningitis, demonstrating neutrophilic pleocytosis, elevated protein levels, and a cloudy appearance. Although CSF cultures remained negative, multiplex NAAT rapidly identified both *S. pneumoniae* and VZV, underscoring the diagnostic value of molecular testing in CNS infections. Early pathogen identification facilitated prompt narrowing of antimicrobial therapy to ceftriaxone and acyclovir. Ceftriaxone provided appropriate coverage for pneumococcal meningitis, while acyclovir was administered to treat VZV CNS infection. The patient's course was further complicated by NSTEMI, reduced left ventricular ejection fraction, and AKI. These complications are well recognized in severe pneumococcal sepsis and likely reflect a combination of systemic inflammation, hemodynamic instability, and end-organ hypoperfusion. They are associated with prolonged hospitalization and increased mortality, emphasizing the need for close multidisciplinary management [8-10].

This case highlights several important clinical lessons, including the possibility of concurrent bacterial and viral CNS infection in adults, the atypical presentation of meningitis that can mimic acute cerebrovascular disease, and the critical role of molecular diagnostics in establishing a timely diagnosis. Early recognition and targeted treatment are essential to improving outcomes in these complex and potentially life-threatening infections.

## Conclusions

In conclusion, this case highlights the diagnostic challenges posed by concurrent bacterial and viral CNS infections and the value of rapid molecular diagnostics, including NAAT and multiplex PCR, for early pathogen identification and targeted therapy. Older adults may present with atypical clinical features, delaying diagnosis, and mixed infections should be considered when the severity of illness exceeds what would be expected from a single pathogen. This case also underscores the potential for severe systemic complications, emphasizing the importance of multidisciplinary management, prompt initiation of appropriate therapy, and pneumococcal vaccination to improve patient outcomes.
